# Effects of Intra-Amniotic Administration of the Hydrolyzed Protein of Chia (*Salvia hispanica* L.) and *Lacticaseibacillus paracasei* on Intestinal Functionality, Morphology, and Bacterial Populations, In Vivo (*Gallus gallus*)

**DOI:** 10.3390/nu15081831

**Published:** 2023-04-11

**Authors:** Marcella Duarte Villas Mishima, Hércia Stampini Duarte Martino, Nikolai Kolba, Drashti Dhirenkumar Shah, Mariana Grancieri, Karina Maria Olbrich Dos Santos, Janine Passos Lima, Bárbara Pereira Da Silva, Elvira Gonzalez de Mejia, Elad Tako

**Affiliations:** 1 Department of Food Science, Stocking Hall, Cornell University, Ithaca, NY 14853, USA; 2 Department of Nutrition and Health, Federal University of Viçosa, Av. Purdue, s/n, Campus Universitário, Viçosa 36570-900, MG, Brazil; 3 Embrapa Agroindústria de Alimentos, Av. das Américas 29.501, Rio de Janeiro 23020-470, RJ, Brazil; 4 Department of Food Science & Human Nutrition, University of Illinois at Urbana-Champaign, Urbana, IL 61801, USA

**Keywords:** gut health, microbiota, intestinal barrier, inflammation, chia seed, bioactive peptides, in vivo, probiotic

## Abstract

As a protein source, chia contains high concentrations of bioactive peptides. Probiotics support a healthy digestive tract and immune system. Our study evaluated the effects of the intra-amniotic administration of the hydrolyzed chia protein and the probiotic *Lacticaseibacillus paracasei* on intestinal bacterial populations, the intestinal barrier, the inflammatory response, and brush border membrane functionality *in ovo* (*Gallus gallus*). Fertile broiler (*Gallus gallus*) eggs (*n* = 9/group) were divided into 5 groups: (NI) non-injected; (H_2_O) 18 MΩ H_2_O; (CP) 10 mg/mL hydrolyzed chia protein; (CPP) 10 mg/mL hydrolyzed chia protein + 10^6^ colony-forming unit (CFU) *L. paracasei*; (P) 10^6^ CFU *L. paracasei.* The intra-amniotic administration was performed on day 17 of incubation. At hatching (day 21), the animals were euthanized, and the duodenum and cecum content were collected. The probiotic downregulated the gene expression of NF-κβ, increased *Lactobacillus* and *E. coli*, and reduced *Clostridium* populations. The hydrolyzed chia protein downregulated the gene expression of TNF-α, increased OCLN, MUC2, and aminopeptidase, reduced *Bifidobacterium*, and increased *Lactobacillus*. The three experimental groups improved in terms of intestinal morphology. The current results suggest that the intra-amniotic administration of the hydrolyzed chia protein or a probiotic promoted positive changes in terms of the intestinal inflammation, barrier, and morphology, improving intestinal health.

## 1. Introduction

Chia (*Salvia hispanica* L.) is considered a potentially bioactive food source since it has demonstrated multiple health benefits, such as a decrease in adiposity, modulation of the glucose metabolism [1], a hypoglycemic effect, a reduction in hepatic fat deposition [2], an improved lipid profile [2,3,4,5], reductions in inflammatory processes [4,5,6], antioxidant protection [4,7] and reduced fat content of the heart [7]. Regarding intestinal health, chia seeds have been shown to improve the intestinal brush border membrane and favored its functionality [2,8,9,10], increasing the production of short-chain fatty acids and increasing the richness of the microbiota [9,10].

Chia seeds have high concentrations of lipids (30.17 g.100 g^−1^), proteins (19.72 g.100 g^−1^), total dietary fiber (37.18 g.100 g^−1^), and bioactive compounds such as phenolic compounds, tocopherols, and tocotrienols [11]. As a protein source, the hydrolyzed chia protein seeds contains high concentrations of bioactive peptides with promising compositions and sequences. The amino acid sequences of chia seed proteins have been identified; these proteins are associated with hypoglycemic and hypotensive activity, an antioxidant effect, and glucose uptake stimulating peptides [12]. Furthermore, the hydrolyzed chia protein demonstrated these antioxidant, antihypertensive, and hypoglycemic properties in vitro [13]; meanwhile, in vivo, it improved the biochemical profile, reduced TNF-α expression, and reduced the production of activated NF-κβ and affected adipogenesis, with antilipidemic and antiadipogenic actions [14]. Bioactive peptides have diverse functions, including modulating intestinal homeostasis and affecting barrier function, the villus surface area, mucosal immune responses, inflammation, and the gut microbiota [15,16,17,18]. Studies are still needed to understand the effects of the intestinal environment on the bioavailability of bioactive peptides, their interactions with other compounds, and fundamental questions regarding the effectiveness of hydrolyzed protein as a source of bioactive peptides on intestinal health, as well as the interactions between intestinal barrier function, microbiota, and the immune system [19].

Probiotics are live microorganisms that, when administered in adequate amounts, confer benefits to the health of the host. Probiotic use supports a healthy digestive tract and a healthy immune system, so the overall benefit of probiotics on the gut microbiota derives from creating a favorable gut environment [20,21,22]. Probiotic food products represent a market trend, being associated with the increasing consumer awareness regarding the link between diet and well-being, and especially gut health. Strains pertaining to several *Lactobacilli* were shown to exhibit probiotic properties, particularly within the *Lacticaseibacillus* species [23,24,25]. *Lactobacilli* are considered autochthonous residents in the gastrointestinal tracts of animals such as chickens, rodents, and humans [24,25]. They may promote the host’s intestinal health and immune function in different ways, such as by strengthening the epithelial barrier, competitively rejecting pathogenic microorganisms, producing antimicrobial substances, and interacting with immune cells by stimulating pattern recognition receptors [24]. Studies have shown that *Lacticaseibacillus paracasei* upregulated the expression of tight junction proteins, downregulated the production of pro-inflammatory cytokines, and altered the structure of the intestinal microbiota [26].

The *Gallus gallus* is an animal model that presents a complex gut microbiota with gene sequences that are, at the phylum level, highly homologous to humans [27]. The intra-amniotic feeding model is widely used as an in vivo method to assess bioactive compounds with potential nutritional effects, specifically prebiotic effects. The intra-amniotic administration is conducted on day 17 of embryonic development, prior to the embryo’s oral consumption of the amniotic fluid [28]. The intra-amniotic administration of proteins can stimulate the maturation and functionality of the small intestine by promoting cell proliferation and differentiation and the expansion of the absorptive surface area [29]. The intra-amniotic administration of duck egg white peptides was able to increase the villus surface area and diameter of goblet cells by promoting the proliferation of enterocytes, promoting beneficial bacterial populations, and limiting potentially pathogenic bacterial populations, in addition to promoting and contributing to calcium uptake [15]. The use of probiotics in poultry nutrition has shown several benefits, such as improvements in the microbiological homeostasis of the intestine, and in the immune response and growth. These benefits vary due to differences differences in, for example, the strains and the doses of the probiotics [30]. The intra-amniotic administration of a probiotic can upregulate the mRNA expression of intestinal-function-related genes and nutrient-transporter-related genes [31].

To date, no studies have explored the combined effects of the intra-amniotic application of hydrolyzed chia protein and the probiotic *L. paracasei*. As such, the objective of this study was to conduct an in vivo assessment of the effects of the intra-amniotic administration of hydrolyzed chia protein and the probiotic *L. paracasei* on intestinal bacterial populations, the intestinal barrier, the inflammatory response, and the brush border membrane’s (BBM) functionality. Bioactive peptides might have functions that affect intestinal homeostasis, in addition to probiotics, which have been demonstrated to play a supporting role in the digestive tract’s health and function; we therefore hypothesized that, combined with the probiotic, the hydrolyzed chia protein would increase the abundance of beneficial bacterial populations. This effect will further improve the intestinal BBM’s functionality via regulation of the gene expression of key proteins that are required for tight junction development and inflammatory responses.

## 2. Materials and Methods

### 2.1. Sample Material

#### 2.1.1. Hydrolyzed Chia protein

Chia seeds grown in Rio Grande do Sul/Brazil were prepared according to Orona-Tamayo (2015) [32]. The total digested protein was produced and analyzed in a Laboratory at the University of Illinois, Urbana-Champaign, IL, USA, as detailed by Grancieri (2019) [13]. Briefly, for mucilage formation, the seeds were immersed for 1 h in distilled water (1 g/20 mL), frozen overnight (−80 °C), and freeze-dried (Labconco Freeze Dryer 4.5; Kansas, MO, USA). The mucilage was removed from the seeds using a sieve (500 μm per 35 mesh). Using a coffee grinder (Mr. Coffee^®^, Cleveland, OH, USA), the mucilage-free seeds were ground, sieved (500 μm per 35 mesh), and degreased using hexane (1 g/10 mL) at 60 °C for 2 h under constant stirring. The mixture was centrifuged (6000× *g*, 15 min, 4 °C) and the resulting flour was left overnight under a hood and stored at 4 °C until use. Then, deionized water was added to the mucilage and fat-free chia flour (1 g/20 mL); the pH was adjusted to 8.0 and it was kept under constant stirring (35 °C per 1 h). The mixture was centrifuged (5000× *g*; 15 min; 25 °C) and the supernatant was collected, freeze-dried, and stored at −20 °C. This fraction is referred to as “total proteins”.

For the digestion of the extracted protein, we used a previously described technique [33] in which gastrointestinal digestion is simulated. Briefly, the extracted protein was suspended in deionized water (1 g/20 mL), the pH was adjusted to 2.0, pepsin was added at a concentration of 1:20 (enzyme:protein), and it was stirred for 2 h at 37 °C. Afterwards, the pH was adjusted to 7.5, pancreatin 1:20 (enzyme:protein) was added, and then the digestion was carried out as above. Finally, the simulated digestion was stopped by placing the mixture in a water bath (75 °C, 20 min); it was then centrifuged twice at 20,000× *g* (4 °C, 15 min). The supernatant was collected (total protein digested) and dialyzed using a 100–500 Da molecular weight exclusion membrane (Spectra/Por^®^, Biotech CE Membrane); it was then lyophilized and stored at −20 °C until use.

#### 2.1.2. Probiotic

Freeze-dried *L. paracasei* (strain TRA038563) was provided by the Brazilian Agriculture Research Corporation (EMBRAPA, Rio de Janeiro, Brazil).

### 2.2. The Intra-Amniotic Administration

Cornish Cross fertile broiler eggs (*n* = 50), obtained from a commercial hatchery (Moyer’s chicks, Quakertown, PA, USA), were incubated under optimal conditions (37 ± 2 °C and 89.6 ± 2% humidity) using Cornell University’s Animal Science poultry farm incubator. All animal protocols were approved by Cornell University’s Institutional Animal Care and Use Committee (protocol code: 2020–0077).

The hydrolyzed chia protein in powdered form was diluted in 18 MΩ H_2_O. The osmolarity of the solution was tested to determine the concentration necessary to maintain an osmolarity value (Osm) of less than 320 Osm; this also ensured that the chicken embryos would not be dehydrated upon the injection of the solution. The intra-amniotic administration followed the methodology previously described [34,35,36].

At 17 days of embryonic incubation, eggs were candled to discard those that presented as infertile, cracked, contaminated, or early dead embryos. Eggs containing viable embryos were weighed and randomly allocated into 5 groups with a similar weight frequency distribution (*n* = 10/group). Briefly, all eggs were disinfected with 70% ethanol. Each group was then injected with the specified solution (1 mL per egg) with a 19 mm gauge needle that was vertically inserted into the amniotic fluid, which was identified by candling.

The 5 groups were assigned as follows: (NI) non-injected; (H_2_O) 18 MΩ H_2_O; (CP) 10 mg/mL (1%) hydrolyzed chia protein; (CPP) 10 mg/mL (1%) hydrolyzed chia protein + 10^6^ colony-forming unit (CFU) *Lacticaseibacillus paracasei* (800 µL of hydrolyzed chia protein + 200 µL of probiotic/egg); (P) 10^6^ CFU *Lacticaseibacillus paracasei.*

After the injections, the injection holes were sealed with cellophane tape and the eggs placed in hatching baskets. Immediately after hatching (21 days), chicks from each treatment group were weighed and then euthanized by CO_2_ exposure. Their duodenum, cecum, and cecum content were collected.

### 2.3. Extraction of the Total RNA from the Duodenum Tissue Samples

According to the manufacture’s protocol (Rneasy Mini Kit, Qiagen Inc., Valencia, CA, USA), the total RNA was extracted from 30 mg of the duodenum (*n* = 5 animals/group) [35,36,37]. The total RNA was eluted in 50 µL of RNase-free water. All steps were carried out under RNase-free conditions. The RNA was quantified by absorbance at 260/280, and the integrity of the 18S ribosomal RNAs was verified using 1.5% agarose gel electrophoresis, followed by ethidium bromide staining. The samples were stored at −80 °C until the analysis.

### 2.4. Real-Time Polymerase Chain Reaction (RT-PCR) and Prime Design

The cDNA was created with a 20 µL reverse transcriptase (RT) reaction in a BioRad C1000 touch thermocycler using the Improm-II Reverse Transcriptase Kit (Catalog #A1250; Promega, Madison, WI, USA). The concentration of cDNA obtained was determined by measuring the absorbance at 260 nm and 280 nm using an extinction coefficient of 33 (for single-stranded DNA). The gene expression of the duodenum was determined using real-time polymerase chain reaction (RT-PCR).

The primers used in the real-time qPCR (Table 1) were designed based on gene sequences from the GenBank database using Real-Time Primer Design Tool software (IDT DNA, Coralvilla, IA, USA) [36,38]. The specificity of the primers was tested by performing a BLAST search against the genomic National Center for Biotechnology Information (NCBI) database. The *Gallus gallus* primer 18S rRNA was designed as a reference gene.

### 2.5. Real-Time qPCR Design

All procedures were performed as previously described [8,34,36,39]. Briefly, cDNA was used for each 10 μL reaction containing 2 × BioRad SSO Advanced Universal SYBR Green Supermix (Hercules, CA, USA). For each reaction, 8 µL of the master mix and 2 µL of cDNA were pipetted into a 96-well plate; meanwhile, for the standard curve, 7 points were evaluated in duplicate. A “no template” control of nuclease-free water was included to exclude DNA contamination in the PCR mix. Table 1 shows the primers used in this study. The double-stranded DNA was amplified using Bio-Rad CFX96 Touch (Hercules, CA, USA) under the following PCR conditions: initial denaturing at 95 °C for 30 s, 40 cycles of denaturing at 95 °C for 15 s, various annealing temperatures according to Integrated DNA Technologies (IDT) for 30 s, and elongating at 60 °C for 30 s.

The data on the expression levels of the genes were obtained as Cp values based on the “second derivative maximum” (automated method) as computed by Bio-Rad CFX Maestro 1.1 (Version 4.1.2433.1219, Hercules, CA, USA). The assays were quantified by including a standard curve in the real-time qPCR analysis, and a standard curve with 4 points was prepared with a 1:10 dilution (duplicates). The software produced a Cp vs. log 10 concentrations graph, and the efficiencies were calculated as 10 (1/slope). The specificity of the amplified real-time RT-PCR products was verified using melting curve analysis (60–95 °C) after 40 cycles, resulting in several different specific products with specific melting temperatures.

### 2.6. Collection of Microbial Samples and DNA Extraction of Intestinal Content

The cecum was removed in a sterile manner and treated as described previously [15,34]. Briefly, the cecum contents were homogenized using a vortex and glass beads. Debris was removed and the supernatant was collected and centrifuged. The pellet was washed and stored at −20 °C until DNA extraction. Then, the pellet was resuspended in ethylenediaminetetraacetic acid (EDTA) and treated with lysozyme (Sigma Aldrich CO., St. Louis, MO, USA; final concentration 10 mg/mL). A Wizard Genomic DNA purification kit (Promega Corp., Madison, WI, USA) was used to isolate the bacterial genomic DNA.

### 2.7. Primer Design and PCR Amplification of Bacterial 16S rDNA

Primers for *Bifidobacterium*, *Lactobacillus*, *Escherichia coli*, and *Clostridium* were used [40]. The universal primers were designed with the invariant region in the 16S rRNA of the bacteria and were used as internal standards. The relative abundance of each examined bacterium was evaluated as previously described [38,41]. Briefly, the PCR products were quantified using 2% agarose gel and stained with ethidium bromide. All products are expressed relative to the content of the universal 16S rRNA primer product and the proportions of each examined bacterial product.

### 2.8. Morphological Examination of Duodenal Tissue

Intestinal morphology was performed as previously described [8,36]. Briefly, the duodenum samples were fixed in fresh 4% (*v*/*v*) buffered formaldehyde; numerous sections were cut and placed on glass slides. The sections were deparaffinized in xylene and rehydrated in ethanol. Afterwards, the slides were stained with Alcian Blue/Periodic acid-Schiff and examined using light microscopy (CellSens Standard software, Olympus, Waltham, MA, USA). The following morphometric measurements were evaluated: villus height (µM), villus surface area (µM), depth of crypts (µM), Paneth cell number and diameter (µM), goblet cell number and goblet cell diameter (µM) in the villi and crypts, and goblet cell type (acidic, neutral, and mixed). In total, 4 segments for each biological sample (*n* = 3/treatment group) were performed and 10 randomly selected villi and crypts were analyzed per segment (40 replicates per biological sample). For the Alcian Blue and Periodic acid-Schiff stain, the segments were only counted for the type of goblet cells (acid, neutral, or mixed) in the villi epithelium and in the crypts. The goblet cells were enumerated at 10 villi or crypts/sample, and the means were calculated for statistical analysis. The *villus surface area* was obtained using the following equation:

(1) $$Villus\;surface\;area=2\frac{VW}{2}\times VL$$

where *VW* = the villus width average of three measurements, and *VL* = the villus length.

A representative image of the histological cross-section of the duodenum from each experimental group indicates the villus surface area measurements.

### 2.9. Statistical Analysis

Experimental treatments for the intra-amniotic administration procedure were arranged in a completely randomized design. All the results were expressed as means ± standard error deviation (SED) from 7 to 9 biological samples per treatment group (according to hatching). Differences were considered significant when *p* < 0.05.

The Shapiro–Wilk normality test was used to evaluate values for normal distribution and variance homogeneity. Normally distributed results were analyzed using a one-way analysis of variance (ANOVA). For a significant “*p*-value,” the post hoc Duncan test was used for those with a normal distribution. The means without normal distribution were analyzed using Kruskal–Wallis and a post hoc Dunn’s test. The statistical analyses were performed using the statistical software IBM SPSS Statistics^®^, version 25.

The correlation between the biomarkers of intestinal health, the bacterial population, and histological parameters was analyzed using Spearman’s rank correlation coefficient. GraphPad Prism^®^ version 9.0 software packages (GraphPad Software Inc., San Diego, CA, USA) were used for graphics.

## 3. Results

### 3.1. Body Weight

The body weight was similar among all the groups compared according to one way ANOVA followed by the post hoc Duncan test: non-injected (35.00 ± 0.82), 18 MΩ H_2_O (35.13 ± 1.09), hydrolyzed chia protein (36.56 ± 0.65), hydrolyzed chia protein + *Lacticaseibacillus paracasei* (34.38 ± 1.28), and probiotic (*Lacticaseibacillus paracasei*) (34.78 ± 0.95).

### 3.2. Effect of the Chia Protein and/or Probiotic on the Gene Expression of Intestinal Inflammation, Intestinal Barrier Proteins, and Brush Border Membrane Functional Proteins

**Figure 1:**
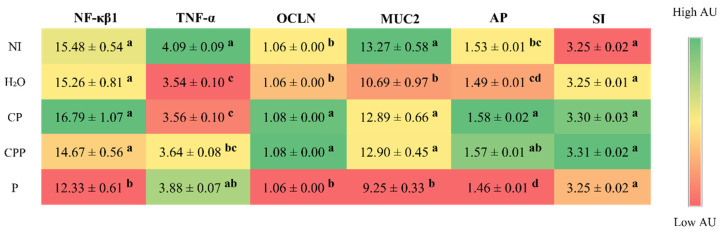
Effect of the intra-amniotic administration of chia protein and/or a probiotic on intestinal (duodenum) gene expression. NI: non-injected; H_2_O: 18 MΩ H_2_O; CP: hydrolyzed chia protein; CPP: hydrolyzed chia protein + *Lacticaseibacillus paracasei*; P: probiotic (*Lacticaseibacillus paracasei*); NF-κβ1: nuclear factor kappa beta; TNF-α: tumor necrosis factor-alpha; OCLN: occludin; MUC2: mucin 2; AP: aminopeptidase; SI: sucrose isomaltase; AU: arbitrary unit. Values are expressed as means ± SEM, *n* = 5/group. Per gene (in the same column), red depicts lower gene expression levels and green depicts higher gene expression levels. ^a–d^ Per gene (in the same column), treatment group means not indicated by the same letter are significantly different (*p* < 0.05) according to one way ANOVA followed by the post hoc Duncan test.

In the duodenum, the expression of tumor necrosis factor alpha (TNF-α) decreased in the groups that received the hydrolyzed chia protein and the hydrolyzed chia protein + a probiotic compared to the non-injected group, but was similar to the group injected with H_2_O. Furthermore, the probiotic group was not able to reduce the expression of TNF-α when compared to both control groups (NI and H_2_O); however, it decreased expression of nuclear factor kappa beta (NF-κβ1) relative to all other groups. Regarding intestinal barrier proteins, the groups that received the hydrolyzed chia protein or the hydrolyzed chia protein + the probiotic showed a higher expression of occluding (OCLN) compared to both control groups (NI and H_2_O); the probiotic group did not show an increase in this parameter. The same occurred for mucin-2 (MUC2); the hydrolyzed chia protein and hydrolyzed chia protein + probiotic groups showed an increase in MUC2 expression, but only in relation to the group injected with H_2_O, and the probiotic group showed no change for this parameter. When we evaluated the expression of intestinal functionality proteins, we observed that the hydrolyzed chia protein group had higher AP expression compared to the control groups (NI and H_2_O), and the hydrolyzed chia protein + probiotic group had higher AP expression in relation to the control group injected with H_2_O; meanwhile, the probiotic group presented AP expression similar to the group injected with H_2_O and lower than that of the other groups. In relation to SI expression, there was no difference between any of the experimental groups (Figure 1).

### 3.3. Effect of Chia Protein and/or a Probiotic on the Bacterial Population in Cecum Contents

**Figure 2:**
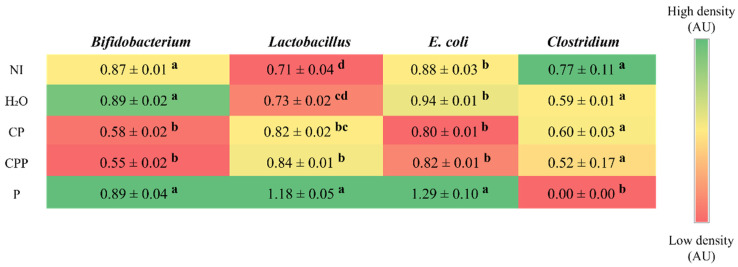
Effect of the intra-amniotic administration of chia protein and/or a probiotic on genera- and species-level bacterial populations from cecal content. NI: non-injected; H_2_O: 18 MΩ H_2_O; CP: hydrolyzed chia protein; CPP: hydrolyzed chia protein + *Lacticaseibacillus paracasei*; P: probiotic (*Lacticaseibacillus paracasei*); AU: arbitrary unit. Values are expressed as means ± SEM, *n* = 5/group. Per bacterial category (in the same column), red depicts lower gene expression levels and green depicts higher gene expression levels. ^a–d^ Per bacterial category (in the same column), treatment group means not indicated by the same letter are significantly different (*p* < 0.05) according to one way ANOVA followed by a post hoc Duncan test.

The intra-amniotic administration of hydrolyzed chia protein and hydrolyzed chia protein + a probiotic reduced the abundance of *Bifidobacterium* compared to both control groups (NI and H_2_O) and the P group. The group probiotic group showed a similar abundance of *Bifidobacterium* to the NI and H_2_O groups. The hydrolyzed chia protein group showed a similar abundance of *Lactobacillus* compared to the H_2_O injection control group and a higher abundance than the NI control group. The abundance of *Lactobacillus* in the hydrolyzed chia protein + probiotic group was similar to the CP and higher than both control groups (NI and H_2_O); additionally, the probiotic group showed a higher abundance of *Lactobacillus* compared to all other groups. Furthermore, the probiotic group showed a higher abundance of *E. coli* compared to all other groups, and all other groups presented a similar abundance of *E. coli*. The abundance of *Clostridium* was similar among the NI, H_2_O, hydrolyzed chia protein, and hydrolyzed chia protein + probiotic groups; it was reduced in the probiotic group (Figure 2).

### 3.4. Effect of Chia Protein and/or a Probiotic on Duodenal Morphological Parameters

**Figure 3:**
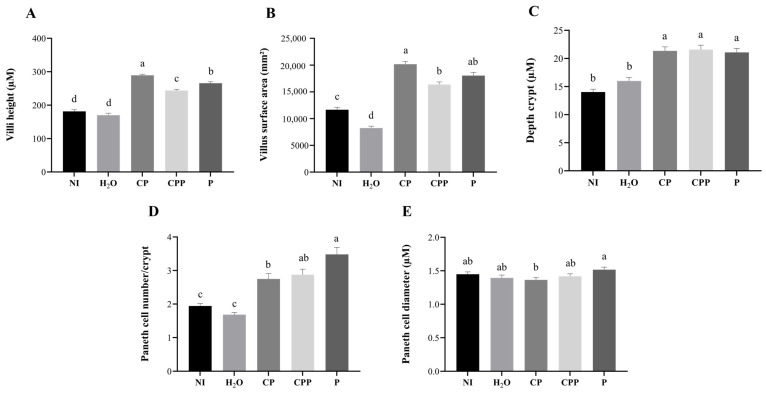
Effect of the intra-amniotic administration of chia protein and/or a probiotic on duodenal morphological parameters. (**A**) Villi height (µM); (**B**) villus surface area (mm^2^); (**C**) crypt depth (µM); (**D**) Paneth cell number/crypt; (**E**) Paneth cell diameter (µM); NI: non-injected; H_2_O: 18 MΩ H_2_O; CP: hydrolyzed chia protein; CPP: hydrolyzed chia protein + *Lacticaseibacillus paracasei*; P: probiotic (*Lacticaseibacillus paracasei*). Values are expressed as means ± SEM, *n* = 3 animals/group, 4 sections, 10 measurements. Treatment group means for specific variables followed by the same letter are not significantly different (*p* > 0.05) according to Kruskal–Wallis and a post hoc Dunn’s test. ^a–d^ Treatment group means not indicated by the same letter are significantly different (*p* < 0.05) according to Kruskal–Wallis and a post hoc Dunn’s test.

**Figure 4:**
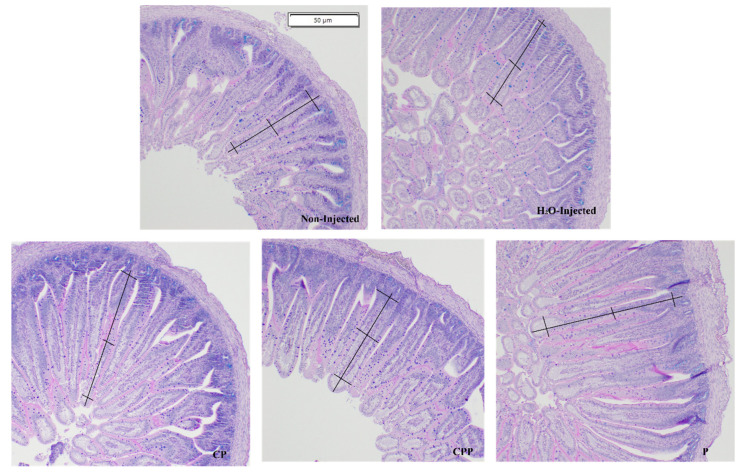
Representation of duodenal cross section morphology for each treatment group. The *villi surface area*, depicted in black, was obtained using the following equation: $Villus\;surface\;area=2\frac{VW}{2}\times VL$, where *VW* = average villus width of three measurements, and *VL* = villus length. NI: non-injected; H_2_O: 18 MΩ H_2_O; CP: hydrolyzed chia protein; CPP: hydrolyzed chia protein + *Lacticaseibacillus paracasei*; P: probiotic (*Lacticaseibacillus paracasei*).

The villi height and villus surface area were increased in all treatment groups (CP, CPP, and P). The hydrolyzed chia protein (CP) group showed villi height and villus surface area higher than all other groups. The hydrolyzed chia protein + probiotic (CPP) group showed villi height and villus surface area higher than both of control groups (NI and H_2_O), and the probiotic (P) group presented villi height greater than both control groups (NI and H_2_O) and CPP group; the villus surface area was higher than both control groups (NI and H_2_O) and similar to the CPP and CP groups (Figure 3 and Figure 4).

The depth of the crypts was increased in all treatment groups (CP, CPP, and P) compared to both of the control groups (NI and H_2_O) and was similar among the experimental groups (Figure 3).

The treatment groups (CP, CPP, and P) had a higher number of Paneth cells compared to the control groups (NI and H_2_O), and the probiotic group had a higher number of Paneth cells compared to the CP group. The Paneth diameter was similar among all the groups, except when comparing the CP and P groups; the administration of the probiotic increased the diameter of Paneth cells compared to those in the group injected with hydrolyzed chia protein (Figure 3).

In the villi, the goblet cell numbers were not different among the treatment groups (CP, CPP, and P); these groups showed numbers of goblet cells similar to the NI group, and lower than the H_2_O group. However, the intra-amniotic administration of hydrolyzed chia protein, CPP, and P increased the diameter of the goblet cells. The CPP and P groups were similar and presented goblet cells with larger diameters compared to both of the control groups (NI and H_2_O). CPP was similar to CP, and P was higher than CP. Similar results were found regarding the diameter of the goblet cells of the crypts. Regarding the goblet cell numbers in the crypt, the H_2_O, CP, CPP, and P groups showed fewer goblet cells than the NI group, and the CPP group had lower numbers than CP and P (Table 2).

In the villi, the treatment groups (CP, CPP, and P) exhibited reduced numbers of acidic and neutral goblet cells and increased numbers of mixed goblet cells. In the crypt, the treatment groups (CP, CPP, and P) reduced neutral goblet cells; for acidic goblet cells, the CP and P groups were similar to both of the controls, and CPP was similar to the H_2_O group. Regarding the mixed goblet cells, the P group was similar to both of the controls and the CP and CPP groups were similar to the H_2_O group (Table 2).

### 3.5. Correlation Analysis

**Figure 5:**
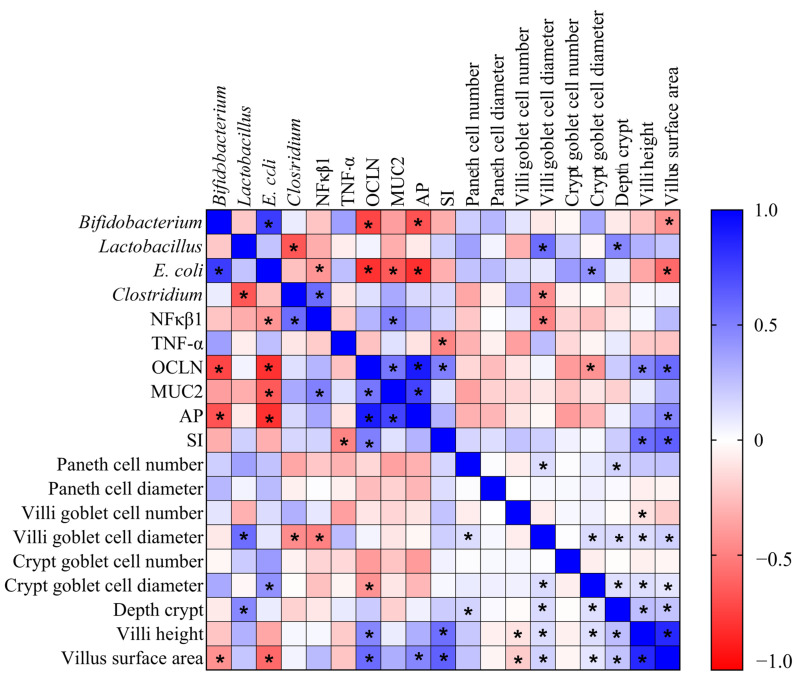
Heatmap of Spearman correlation analysis. NF-κβ1: nuclear factor kappa beta; TNF-α: tumor necrosis factor alpha; OCLN: occludin; MUC2: mucin 2; AP: aminopeptidase; SI: sucrose isomaltase. * Indicates a statistically significant difference (*p* < 0.05).

Spearman correlation analysis was used to assess the relationships between the intestinal parameters investigated in this study, and significant correlations were observed. Positive correlations were observed between OCLN and MUC2, as well as between AP and the villi surface area. Furthermore, negative correlations were observed between *E. coli* and OCLN, MUC2, AP, and the villi surface area (Figure 5).

## 4. Discussion

Chia is a seed with a rich nutritional composition; it is a good source of bioactive peptides [11,12]. However, the potential effects of chia seeds’ hydrolyzed proteins in combination with the probiotic *Lacticaseibacillus paracasei* on intestinal bacterial populations, the intestinal barrier, inflammatory responses, and BBM functionality in vivo have not been investigated. Our results demonstrated that the intra-amniotic administration of the probiotic downregulated NF-κβ1 gene expression, increased the cecal *Lactobacillus* and *E. coli* populations, decreased *Clostridium*, and increased the diameter of goblet cells (Figure 1 and Figure 2 and Table 2). Furthermore, hydrolyzed chia protein downregulated TNF-α expression; moreover, the gene expression of OCLN, MUC2, and AP were improved in the presence of the hydrolyzed protein, either alone or with the probiotic. Although the hydrolyzed chia protein decreased the number of goblet cells, their diameter was increased. The villi height, villi surface area, crypt depth, and number of Paneth cells were increased in all treatment groups (CP, CPP, and P) compared to the control groups (NI and H_2_O).

In the present study, administration of the probiotic downregulated NF-κβ1 gene expression compared to both of the control groups (NI and H_2_O) and to the other treatment groups (CP and CPP). Moreover, TNF-α expression decreased in the groups administered the hydrolyzed chia protein (Figure 1). NF-κβ1 is responsible for inducing cytokine gene expression to control inflammatory and immune responses [42]. NF-κβ is translocated to the cytosol and into the nucleus to initiate the pathway to drive the expression of target genes such as TNF-α, IL1β, IL6, and IL10 [43]. The hydrolyzed chia protein also demonstrated effects in reducing the secretion of TNF-α in an in vitro study and showed effects in the adipose tissue in an in vivo study [14,44]. These observations suggest that the probiotic has an anti-inflammatory effect, as was previously reported [20,45]. Likewise, the hydrolyzed chia protein might have similar effects, as demonstrated in the current study. Probiotic bacteria play a role in the regulation of the immune response by stimulating immune cells through the modulation of intestinal microbiota and downregulating inflammation [21,22]. Some of these bacteria might be useful in mitigating intestinal inflammation; specifically, *Lactobacillus* and *Bifidobacteria* demonstrate antimicrobial effects by affecting both local and systemic immune responses [20,45]. Peptides with hydrophobic amino acids have more ionizable groups that block free radicals and then increase antioxidant activity [46]. This activity was also observed in peptides with fewer than 20 amino acid residues per molecule, since small peptides are better able to cross the intestinal barrier and exert their biological effects [47]. Furthermore, peptides with hydrophobicity ≤20 kcal mol^−1^ are more effective at penetrating the cell membrane and exercising effects on the molecule [48]. Most of the peptides found in our hydrolyzed chia protein showed these characteristics, which may explain their beneficial effects against inflammation [12,13]. The main storage protein fractions found in chia seeds are albumin, globulin, glutelin, and prolamin [12,13].

Intestinal inflammation is associated with impaired barrier function, leading to increased intestinal permeability and bacterial translocation [43]. In our study, both groups administered hydrolyzed chia protein (CP and CPP) showed increased gene expression of OCLN and MUC2 compared to the H_2_O-injected control group. TNF-α is a pro-inflammatory cytokine that plays a key role in the inflammatory cascade that causes increased intestinal epithelial permeability and then leads to chronic intestinal inflammation [26,49,50]. Thus, as this study found, the bioactive peptides present in hydrolyzed chia protein can reduce the disruption of the intestinal barrier by reducing inflammatory factors such as TNF-α [51].

The mucus layer acts as the first barrier on the surface of the gastrointestinal tract; it is an innate host defense mechanism [52]. Mucus is essential for regulating the homeostasis of the intestinal microbiota and preventing disease, which it does by protecting the gastrointestinal barrier from pathogenic microorganisms, toxins, and other irritants [53,54,55]. MUC2 is the main mucin specifically produced in goblet cells [52]. Under intestinal inflammatory conditions, with a reduction in goblet cells, the expression of MUC2 is reduced, and the mucus loses its barrier function and exposes the mucous membrane to inflammatory substances, such as pathogenic microorganisms, toxins, and lipopolysaccharides [52,55]. We observed that hydrolyzed chia protein, either alone or when combined with a probiotic, increased the gene expression of MUC2; this is probably due to the increased diameter of the goblet cells, which improves the protection of the intestinal barrier. As the tight junction is interdependent with the mucus barrier, the loss of one can reduce the other; this interdependence may result from the regulation of signals that regulate both the mucus and the tight junction [52]. Based on the correlation analysis (Figure 5), the gene expression of OCLN was positively correlated with the gene expression of MUC2. Moreover, with the increase in OCLN and MUC2, the intestinal barrier can protect the host against the permeability of pathogenic components.

Aminopeptidase (AP) is an exopeptidase that cleaves amino acids from the N-terminus of peptides; it is located on the enterocyte’s BBM [28]. The upregulation of the BBM and functional genes’ expression reflects intestinal development and digestive capabilities. Thus, it also affects the potential increased absorption of nutrients [28,56]. In the current study, AP gene expression was upregulated, probably due to the increase in the villi height and villi surface area, which is accordance with our correlation analysis. This increase in the villi surface area leads to more enterocytes and, consequently, higher functionality and absorption capacity [56]. This improvement in absorption capacity is important for the absorption of peptides that play an anti-inflammatory role. In addition, the administration of CP, CPP, and P maintain the gene expression of sucrose isomaltase (SI), compared to the non-injected and H_2_O-injected groups (Figure 1).

The intra-amniotic administration of hydrolyzed chia protein, hydrolyzed chia protein + probiotic or just the probiotic (*L. paracasei*) were able to increase villi height, villi surface area and crypt depth, but the increases were higher when these compounds were administered separately (Figure 3). The intestinal villi play an essential role in the digestion and absorption of nutrients, as the villi increase the internal surface area and, in turn, the digestive and absorptive capacities of the BBM [8,28,56]. The crypts are comprised of continuously proliferating stem cells; these are responsible for the formation of enterocytes, which play a key role due to their nutrients’ absorptive ability [57]. Deeper crypts lead to an increase in the secretion of digestive enzymes [57]. Thus, the surface area of the villi and the crypts’ depth are indictors of intestinal developmental and functional status. Therefore, an increase in these morphometric parameters due to CP, CPP, or P can improve the digestive and absorptive capabilities of the BBM [28], as demonstrated by the correlation analysis (Figure 5).

Paneth cells are found in the crypts; they are secretory cells that produce antimicrobial peptides, proteins, and other important components of host defense and immunity [58]. Dysfunction of Paneth cells can disrupt these functions, leading to an imbalance in the gastrointestinal tract. Decreased numbers of Paneth cells, followed by the leakage of bacteria, are often seen in various infectious diseases [58]. Here, we found that, in the treatment groups (CP, CPP, and P), the number of Paneth cells, but not their diameter, was increased; therefore, we suggest that the intra-amniotic administration of hydrolyzed chia protein, hydrolyzed chia protein + a probiotic, or just the probiotic (*L. paracasei*) improved Paneth cell development without affecting the size of the cells. This indicates that the antimicrobial peptides secreted by Paneth cells were not produced, since there were no stimuli such as inflammation or pathogenic bacteria. Similar results were demonstrated after the intra-amniotic administration of black corn soluble extract, a source of phenolics [34].

Goblet cells produce mucin, the most important substance in the mucus layer, which forms a gel barrier against pathogenic bacteria [54]. Our results showed that the investigated probiotic increased the diameter of goblet cells; meanwhile, the intra-amniotic administration of hydrolyzed chia protein + a probiotic decreased the number of goblet cells, but their diameter was increased. It is possible that the observed reduction in the inflammatory biomarker (TNF-α) may have led to an increased goblet cell diameter and, therefore, increased MUC2 gene expression. Regarding the types of goblet cells, the treatment groups (CP, CPP, and P) reduced the number of acidic goblet cells in the villi. An acidic pH in the intestine facilitates the growth of beneficial bacteria over detrimental bacteria [59]. Therefore, the reduction in acidic goblet cells might be associated with the increase in the *E. coli* population, as seen in the probiotic group [60].

The microbial analysis revealed that the probiotic *L. paracasei* increased the abundance of *Lactobacillus* and *E. coli* and reduced *Clostridium* populations in the cecal contents, in comparison to all the other groups (Figure 2). Furthermore, the intra-amniotic administration of hydrolyzed chia protein and hydrolyzed chia protein + a probiotic *(L. paracasei*) reduced the abundance of *Bifidobacterium* but increased *Lactobacillus*. *Lactobacillus* species are considered probiotics due to their immunomodulatory and anti-inflammatory actions, their competition with pathogens, and their stimulation of the release of antimicrobial substances, especially mucin, which activates the MUC2 gene and prevents pathogens from adhering to the epithelial barrier [45]. In contrast, the E. coli genus may impair the epithelial barrier by disrupting tight junction proteins [53], as indicated by the negative correlation between *E. coli* and OCLN (Figure 5). The intra-amniotic administration of the probiotic (*L. paracasei*) did not improve the intestinal barrier, as we found in the group administered the hydrolyzed chia protein; this finding might be related to the increase in *E. coli*. Mucus is an important barrier against potentially pathogenic bacteria [52,55]. In the probiotic group, the *E. coli* population was increased and MUC2 gene expression remained constant; however, when the hydrolyzed chia protein was added to this probiotic, MUC2 expression was upregulated, and *E. coli* abundance remained similar (*p* > 0.05) to that seen in the control groups. Therefore, it is likely that the presence of the hydrolyzed chia protein can increase MUC2 and prevent the increase in *E. coli*.

It is important to highlight that, in this study, we investigated the administration of only one strain of a probiotic, but the literature highlights the importance of administering different strains of probiotics, to obtain greater benefits [20]. The *Lacticaseibacillus paracasei* used in this study is an innovative culture to be tested on plant products without fermentation, which has a promising probiotic effect.

**Figure 6:**
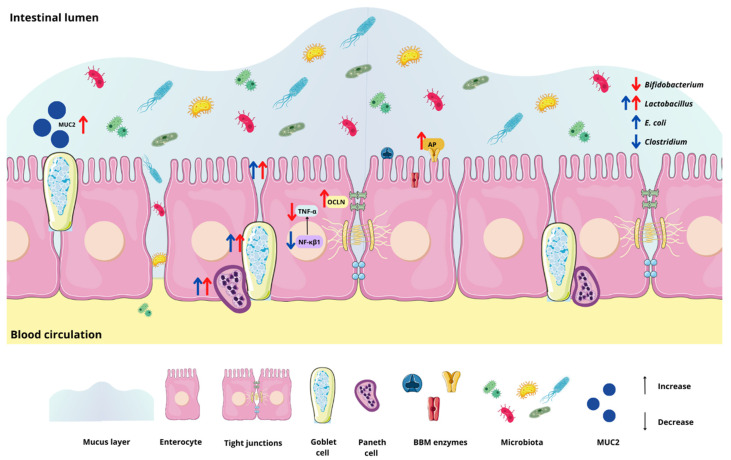
Graphical representation of the results. The intra-amniotic administration of hydrolyzed chia protein reduced TNF-α and the *Bifidobacterium* population and increased OCLN, MUC2, AP, the *Lactobacillus* population, villi height, villi surface area and crypt depth, the Paneth cell number, and the diameter of goblet cells. The administration of the probiotic reduced NF-κβ1 and *Clostridium* populations, and increased *Lactobacillus* and *E. coli*, the villi height, villi surface area, and crypt depth, the Paneth cell number, and the diameter of the goblet cells. NF-κβ1: nuclear factor kappa beta; TNF-α: tumor necrosis factor alpha; OCLN: occludin; MUC2: mucin 2; AP: aminopeptidase; red arrow: effects of hydrolyzed chia protein; blue arrow: effects of *L. paracasei*.

The administration of hydrolyzed chia protein and the administration of the probiotic (*L. paracasei*) were able to improve intestinal health, and we found more pronounced effects when they were administered separately. When the probiotic is administered with the protein, it can protect the probiotic during digestion, and it can arrive in the intestine in a more intact state or in different quantities. This may be the reason for the differences in effects when the compounds were administered alone or together. The probiotic had effects on inflammation and microbiota composition, the hydrolyzed chia protein had effects on inflammation, barrier function, and functionality, and both had an impact on intestinal morphology. Thus, the administration of both can bring about benefits for intestinal health (Figure 6). The current study is the first to investigate the intestinal effects of hydrolyzed chia protein and the probiotic *Lacticaseibacillus paracasei* in vivo; thus, future studies that aim to assess the effects in a long-term feeding trial should be conducted to better understand these effects, since the hydrolyzed chia protein and the probiotic *L. paracasei* presented good results in relation to the improvement of intestinal health.

## 5. Conclusions

The intra-amniotic administration of hydrolyzed chia protein decreased TNF-α, increased the *Lactobacillus* population, OCLN, MUC2, and AP, increased the villi height, villi surface area, and crypt depth, and increased the Paneth cell number and goblet cell diameter. The administration of the probiotic (*L. paracasei*) promoted NF-κβ1 reduction, increased *Lactobacillus* and reduced *Clostridium*, and increased the villi height, villi surface area, crypt depth, Paneth cell number, and goblet cell diameter. Hydrolyzed chia protein and the probiotic (*L. paracasei*) modulate some aspects of intestinal health, and we found more pronounced effects when they were administered separately. These findings suggest that the intra-amniotic administration of hydrolyzed chia protein or a probiotic (*L. paracasei*) improved intestinal health. Therefore, the current study indicates that additional long-term in vivo feeding trials are now warranted to further investigate the observed effects of dietary hydrolyzed chia protein and the experimental probiotic.

## Figures and Tables

**Table 1:** Sequence of experimental primers used in this study.

Gene	Oligonucleotides (5′-3′)	
	Forward Primer (5′-3′)	Reverse Primer (5′-3′)
*BBM functionality*		
AP	CGTCAGCCAGTTTGACTATGTA	CTCTCAAAGAAGCTGAGGATGG
SI	CCAGCAATGCCAGCATATTG	CGGTTTCTCCTTACCACTTCTT
18S rRNA	GCAAGACGAACTAAAGCGAAAG	TCGGAACTACGACGGTATCT
*Inflammation*		
TNF-α	GACAGCCTATGCCAACAAGTA	TTACAGGAAGGGCAACTCATC
NF-κβ1	CACAGCTGGAGGGAAGTAAAT	TTGAGTAAGGAAGTGAGGTTGAG
*Intestinal Barrier*		
MUC2	CCTGCTGCAAGGAAGTAGAA	GGAAGATCAGAGTGGTGCATAG
OCLN	GTCTGTGGGTTCCTCATCGT	GTTCTTCACCCACTCCTCCA

BBM: brush border membrane; AP: aminopeptidase; SI: sucrose isomaltase; TNF-α: tumor necrosis factor-alpha; NF-κβ1: nuclear factor kappa beta; MUC2: mucin 2; OCLN: occludin.

**Table 2:** Effect of chia protein and/or a probiotic on goblet cells.

	NI	H_2_O	CP	CPP	P
Villi Goblet Cell Number	24.68 ± 0.74 ^b^	38.38 ± 0.91 ^a^	26.40 ± 0.72 ^b^	27.46 ± 0.71 ^b^	25.79 ± 0.83 ^b^
Villi Goblet Cell Diameter (μM)	2.45 ± 0.06 ^c^	2.20 ± 0.05 ^d^	2.60 ± 0.06 ^bc^	2.94 ± 0.10 ^ab^	3.13 ± 0.08 ^a^
*Villi Goblet Cell Type Number*					
Acidic	15.28 ± 0.71 ^b^	26.71 ± 1.12 ^a^	10.29 ± 0.59 ^c^	8.49 ± 0.43 ^c^	8.82 ± 0.52 ^c^
Neutral	0.79 ± 0.13 ^a^	0.10 ± 0.04 ^b^	0.28 ± 0.09 ^b^	0.43 ± 0.12 ^b^	0.47 ± 0.12 ^b^
Mixed	8.68 ± 0.57 ^c^	11.57 ± 0.66 ^a^	15.83 ± 0.66 ^a^	18.54 ± 0.65 ^a^	16.51 ± 0.73 ^a^
Crypt Goblet Cell Number	12.67 ± 0.55 ^a^	10.95 ± 0.62 ^b^	10.03 ± 0.36 ^b^	8.04 ± 0.38 ^c^	10.18 ± 0.40 ^b^
Crypt Goblet Cell Diameter (μM)	2.92 ± 0.05 ^d^	3.13 ± 0.05 ^cd^	3.20 ± 0.06 ^bc^	3.40 ± 0.06 ^ab^	3.62 ± 0.07 ^a^
*Crypt Goblet Cell Type Number*					
Acidic	8.53 ± 0.43 ^a^	7.88 ± 0.51 ^ab^	7.63 ± 0.30 ^a^	5.98 ± 0.30 ^b^	7.14 ± 0.33 ^ab^
Neutral	0.41 ± 0.06 ^a^	0.50 ± 0.07 ^a^	0.09 ± 0.03 ^b^	0.08 ± 0.03 ^b^	0.04 ± 0.02 ^b^
Mixed	3.73 ± 0.27 ^a^	2.58 ± 0.21 ^bc^	2.33 ± 0.15 ^bc^	1.98 ± 0.17 ^c^	3.00 ± 0.17 ^ab^

NI: non-injected; H_2_O: 18 MΩ H_2_O; CP: hydrolyzed chia protein; CPP: hydrolyzed chia protein + *Lacticaseibacillus paracasei*; P: probiotic (*Lacticaseibacillus paracasei*). Values are expressed as means ± SED, *n* = 3 animals/group, 4 sections, 10 measurements. ^a–d^ Treatment group means not indicated by the same letter are significantly different (*p* < 0.05) according to Kruskal–Wallis and a post hoc Dunn’s test.

## Data Availability

Not applicable.
